# Impact of Chronic Use of Antimalarials on SARS-CoV-2 Infection in Patients With Immune-Mediated Rheumatic Diseases: Protocol for a Multicentric Observational Cohort Study

**DOI:** 10.2196/23532

**Published:** 2020-10-14

**Authors:** Ana Gomides, Gilda Ferreira, Adriana Kakehasi, Marcus Lacerda, Cláudia Marques, Licia Mota, Eduardo Paiva, Gecilmara Pileggi, José Provenza, Edgard Reis-Neto, Vanderson Sampaio, Ricardo Xavier, Marcelo Pinheiro

**Affiliations:** 1 University Center of Brasília Brasília Brazil; 2 Federal University of Minas Gerais Belo Horizonte Brazil; 3 Universidade do Estado do Amazonas Manaus Brazil; 4 Federal University of Pernambuco Recife Brazil; 5 University of Brasília Brasília Brazil; 6 Federal University of Parana Curitiba Brazil; 7 Faculdade de Ciências da Saúde de Barretos Barretos Brazil; 8 Sociedade Brasileira de Reumatologia São Paulo Brazil; 9 Federal University of São Paulo São Paulo Brazil; 10 Federal University of Rio Grande do Sul Porto Alegre Brazil

**Keywords:** COVID-19, SARS-CoV-2, coronavirus, antimalarial, rheumatic diseases, mortality, immune system, immunology, protocol, observational, pharmacological, drug

## Abstract

**Background:**

COVID-19, caused by the virus SARS-CoV-2, has brought extensive challenges to the scientific community in recent months. Several studies have been undertaken in an attempt to minimize the impact of the disease worldwide. Although new knowledge has been quickly disseminated, including viral mechanisms, pathophysiology, and clinical findings, there is a lack of information on the effective pharmacological management of this disease. In vitro studies have shown some benefits related to the use of antimalarials (chloroquine and hydroxychloroquine) for inhibiting SARS-CoV-2. However, the data from open clinical trials on COVID-19 patients are controversial.

**Objective:**

We present the protocol for a research project that compares the potential protective effect of antimalarials in preventing moderate-to-severe forms of COVID-19 in two groups: (1) patients treated chronically with antimalarials for rheumatic diseases and (2) other members of the patients’ household who have not been diagnosed with rheumatic diseases and are not taking antimalarials.

**Methods:**

This is a 24-week, prospective, observational cohort study comprising patients from public and private health services across Brazil, who chronically use antimalarials for the treatment of immune-mediated rheumatic diseases, osteoarthritis, or chikungunya-related arthropathy. A total of six sequential phone interviews were scheduled during the COVID-19 outbreak in five different regions of Brazil. Information regarding social, epidemiological, and demographic data, as well as details about rheumatic diseases, antimalarials, comorbidities, and concomitant medication, is being recorded using a specific online form in the REDCap database. Symptoms suggestive of COVID-19, including fever, cough, dyspnea, anosmia, and dysgeusia, are being self-reported and collected via phone interviews. Our main outcomes are hospitalization, need of intensive care unit, and death.

**Results:**

Recruitment began at the end of March 2020, and the inclusion was done during an 8-week period (from March 29 to May 17) with a total of 10,443 individuals enrolled at baseline, 5166 of whom have rheumatic diseases, from 23 tertiary rheumatology centers across 97 Brazilian cities. Data analysis is scheduled to begin after all inclusion data have been collected.

**Conclusions:**

This study, which includes a large sample of chronic antimalarial users, will allow us to explore whether SARS-CoV-2 infection may be associated with immune-mediated rheumatic diseases and long-term antimalarial usage.

**Trial Registration:**

Brazilian Registry of Clinical Trials RBR–9KTWX6; http://www.ensaiosclinicos.gov.br/rg/RBR-9ktwx6/

**International Registered Report Identifier (IRRID):**

DERR1-10.2196/23532

## Introduction

COVID-19, which originated from Wuhan, China, in December 2019, remains a major challenge for scientists and the medical community as it continues to spread rapidly across the world [1,2]. The rapid transmission of the disease, which is caused by the novel coronavirus SARS-CoV-2, and the need to minimize its impact have caused scientific research and information to emerge at a record speed, but there are still numerous knowledge gaps [3-6].

In recent months, a marked information revolution has been observed, relating to viral mechanisms, pathophysiology, and heterogeneous clinical findings with different severity grades. Those infected with COVID-19 range from asymptomatic individuals to critically ill patients with outcomes like severe acute respiratory syndrome, coagulopathy (a prothrombotic state triggered by inflammation and other factors), and death [7-13]. Age and concomitant diseases, especially hypertension, diabetes, and heart, kidney, and lung diseases, are associated with poor outcomes [14-18].

Considering there is no specific, effective pharmacological treatment for COVID-19, several drugs have been tested, such as antivirals (lopinavir-ritonavir, remdesivir, favipiravir); antimalarials alone or combined with azithromycine; interleukin 6 (IL-6) antagonists; Janus kinase inhibitors and interferon, as well as other procedures (extracorporeal membrane oxygenation and convalescent plasma). However, to date, there are no data on the potential preventive effect of any of these, regardless of timing (pre-exposure, symptomatic period, or inflammatory phase) [19-27].

The role of chloroquine and hydroxychloroquine in treating patients with malaria and immune-mediated rheumatic diseases (IMRD) is well-known, especially in cases of systemic lupus erythematous and rheumatoid arthritis. In vitro studies have shown antimalarials have an antiviral effect against some viruses, such as SARS-CoV (severe acute respiratory syndrome–associated coronavirus), MERS-CoV (Middle East respiratory syndrome coronavirus), SARS-CoV-2, HIV, Zika, and influenza A(H5N1), especially in relation to endosomal membrane pH changes and an inhibitory mechanism to hamper the viral entry inside the cells. However, clinical trials are controversial and present many methodological problems, including randomization, dosage, time of use, endpoint definitions, outcomes, and safety issues [28-35].

Among rheumatologists, antimalarials have been used safely and effectively for several decades in patients with rheumatic diseases [36]. For this reason, rheumatologists and rheumatic patients who are long-term users of antimalarials were involved in COVID-19–related discussions and research [37-39]. Our main hypothesis is that patients with rheumatic diseases who chronically use antimalarials could have a lower rate of moderate-to-severe forms of COVID-19 since these drugs work as immune modulators in mitigating cytokine storm and poor prognosis.

### Objectives

Our primary aim is to assess the potential preventive effects of antimalarials in reducing the incidence of moderate-to-severe forms of COVID-19 in patients with rheumatic diseases. Secondarily, we aim to determine the frequency of SARS-CoV-2 infection in patients with rheumatic diseases who are chronic users of antimalarials. For both objectives, patients with rheumatic diseases will be compared with members of their household who are not taking antimalarials.

## Methods

### Design

The study design will be a prospective, multicentric, observational cohort study with a control group.

### Sample Size

Considering a moderate-to-severe COVID-19 rate as the dependent variable, a rate which the current literature estimates to be 20% [4,6,10-18,40-42] as well as the proportion of 1 case for every 2 controls, the sample calculation was approximately 3000 antimalarial users and 6000 nonusers, with an error α=5% and β=20%.

### Inclusion Criteria

The inclusion criteria are as follows:

- Men and women;
- >18 years of age;
- Use of antimalarials for at least 30 days before inclusion in the study;
- Diagnosis of IMRD (according to the criteria of the American College of Rheumatology or European League Against Rheumatism), rheumatoid arthritis [43], systemic lupus erythematosus [44], Sjögren's syndrome [45], systemic sclerosis [46], inflammatory myopathies [47], and mixed connective tissue disease [48];
- Diagnosis of osteoarthritis (subgroup) [49];
- Diagnosis of chikungunya-related arthropathy (subgroup).

### Exclusion Criteria

The exclusion criteria, which apply to both patients and controls, are as follows:

- Previous use of chloroquine or equivalent that was not in the past 6 months;
- History of solid organ or bone marrow transplantation;
- Neoplasm of solid organs or lymphatic or myeloproliferative lineage in the past 12 months with or without adjuvant chemotherapy;
- Positive HIV status, regardless of highly active antiretroviral therapy;
- Use of intravenous human immunoglobulin in the past 30 days;
- End-stage renal disease on peritoneal dialysis and hemodialysis.

### Control Group

The control group will consist of healthy individuals aged >18 years, who are household cohabitants or work colleagues of patients with rheumatic diseases in the intervention group.

Contact with a suspected or confirmed case of COVID-19 is defined as an individual sharing accommodations with a patient with a rheumatic disease (eg, residing in the same house or environment as roommates, occupational colleagues, etc). The exposure grade assessment is individualized, considering the type of environment and the exposure time.

For each case, two household and/or professional contact individuals will be selected as controls. The choice of the first control is prioritized by flu-like symptoms and defined as the “symptomatic group,” including suspected or confirmed cases. Those without symptoms will comprise the “asymptomatic group;” both household and/or contact individuals can be selected, preferably paired for age and sex.

The household and/or workplace contacts were chosen to characterize the control group because of their high epidemiological value during community viral transmissions worldwide, instead of including nonusers of antimalarials with rheumatic diseases. Moreover, lupus patients without antimalarial treatment are quite uncommon, except in those with previous toxicity (maculopathy, allergy, long-term remission, etc).

### Data Collection

Data are being collected regarding social, epidemiological, and demographic characteristics, as well as detailed information on antimalarials (type of salt, dosage, frequency, adherence during the pandemic) and rheumatic disease (diagnosis, disease activity). In addition, aspects related to comorbidities, smoking, alcohol intake, and concomitant medications are being recorded, as well as specific information about COVID-19 symptoms and main outcomes (hospitalization, need for intensive care unit, and death) in both groups (Multimedia Appendix 1).

**Figure 1:**
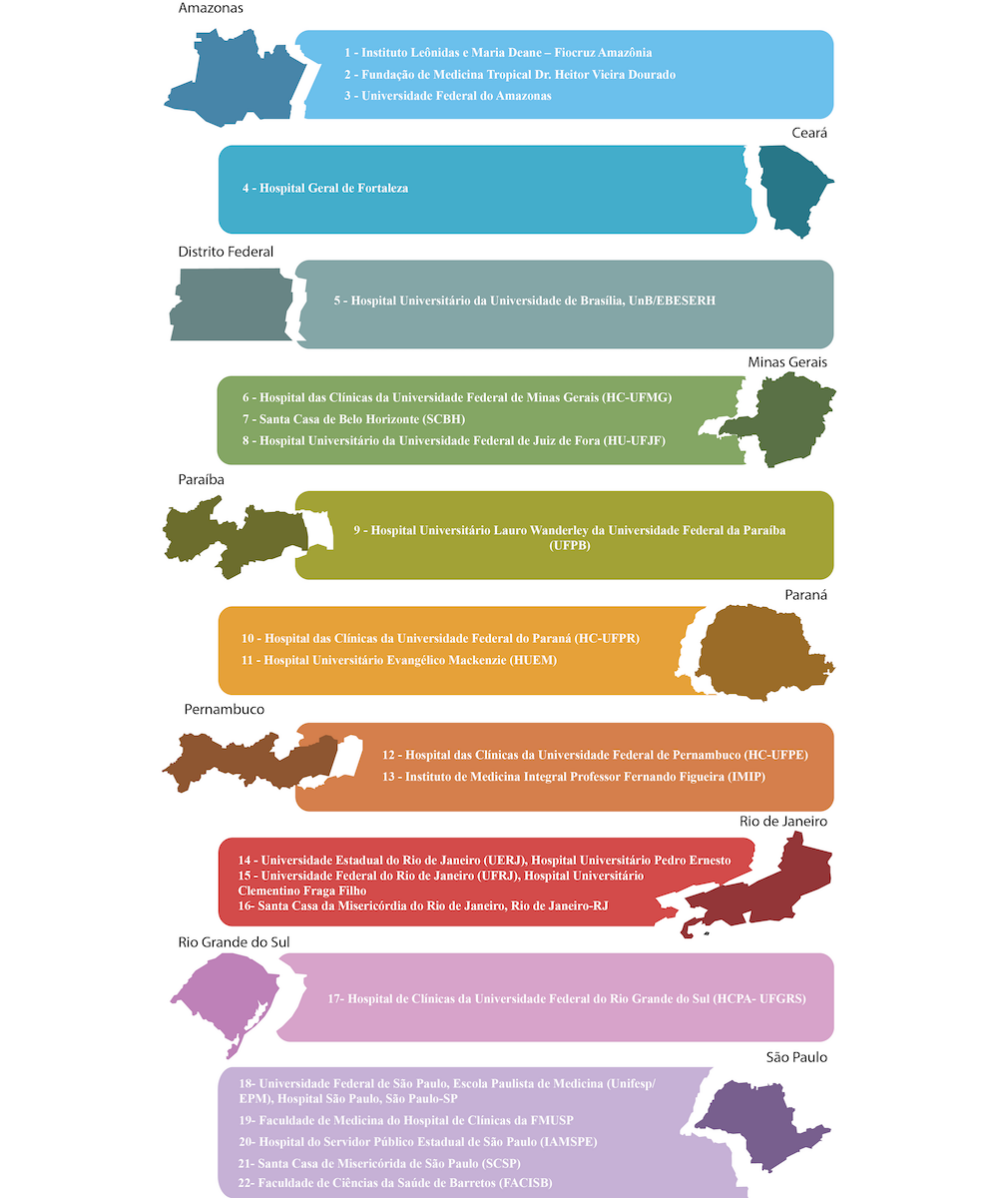
Centers participating in the study.

All phone interviews are being conducted by health care professionals (eg, volunteer medical students), all previously trained by the principal investigator or subinvestigator from each rheumatology center (Figure 1). This training included a tutorial in PDF and video formats about each step of the study protocol. In addition, several WhatsApp-based groups were formed and supervised by the principal investigator, subinvestigator, or study coordinators to find the best solution for minimizing eventual problems.

All patients and controls gave a verbal informed consent before participating in this cohort study. The need for signed informed consent was waived by the Brazilian National Ethics Committee (CONEP) due to the urgency of the pandemic. However, it is worth emphasizing that participation was voluntary for both groups. Research subjects will not be identified by their full name at any time and will not have their personal data disclosed.

**Figure 2:**
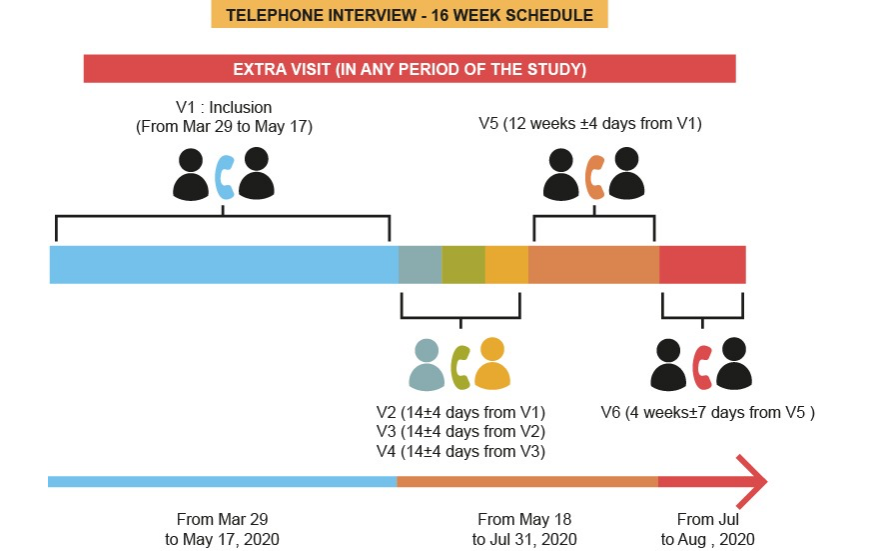
Telephone interview schedule.

The data are being stored on the REDCap platform, with telephone interviews performed as shown in Figure 2. Interviews with patients with invalid contact information or who did not answer the telephone after three consecutive calls at intervals of 3 days were canceled, although patients were not removed from the study. All patients will be evaluated during the closing visit (V6), unless they decline to participate, regardless of whether intermediary visits were missed (V2 to V5).

**Figure 3:**
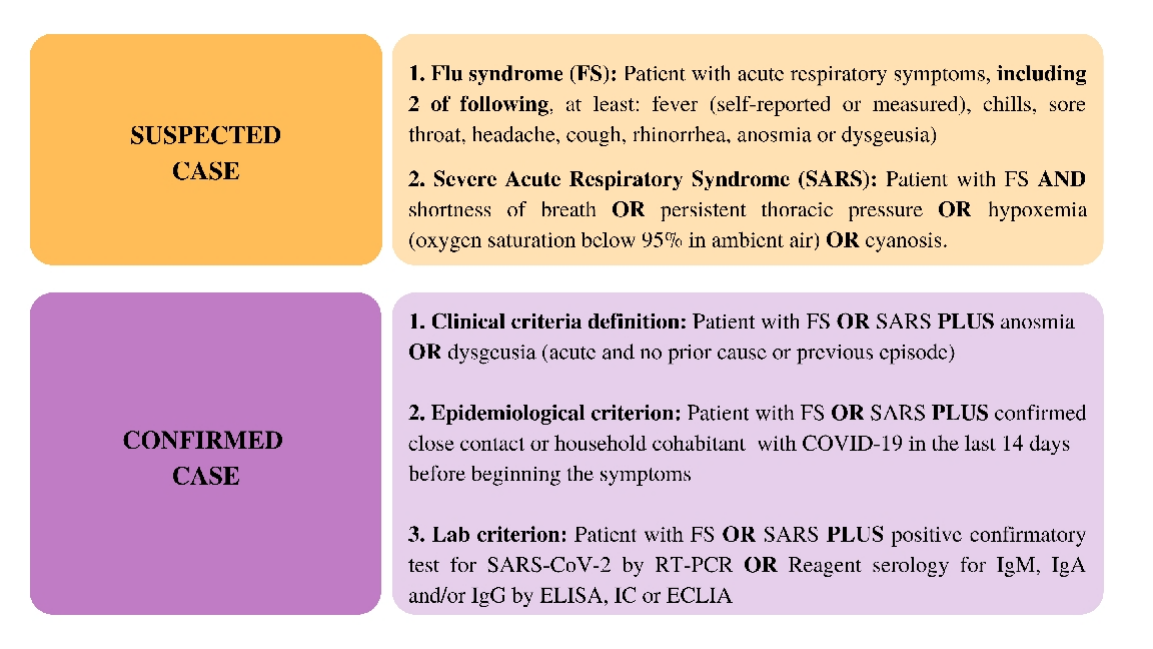
Suspected vs confirmed cases of COVID-19. RT-PCR: real-time polymerase chain reaction, IgM: immunoglobulin M, IgA: immunoglobulin A, IgG: immunoglobulin G, ELISA: enzyme-linked immunosorbent assay, IC: ion channel, ECLIA: electrochemiluminescence immunoassay.

A clinically confirmed case of COVID-19 was defined according to the Brazilian Ministry of Health criteria [50] that included self-reported symptoms (fever, cough, nasal congestion, shortness of breath, malaise, myalgia, decline in general condition, and sudden anosmia and/or dysgeusia) and/or have been in contact with someone with a confirmed or suspected case of COVID-19. A positive PCR (polymerase chain reaction) test for SARS-CoV-2, via oropharyngeal and nasopharyngeal sampling or a specific confirmatory serology (immunoglobulin G and/or immunoglobulin M), is needed to test for infection, according to the Brazilian Ministry of Health [50] (Figure 3).

Moderate-to-severe cases are defined as those that require hospitalization, mechanical ventilation, or result in death. Through direct contact with hospitals, hospitalized patients are being clinically evaluated in terms of longitudinal follow-up and treatment outcome, infection severity, length of stay, need for intensive care, mechanical ventilation, and cause of death.

Considering the heterogeneous community viral transmission in Brazil, some epidemiological approaches were developed to assure a similar path involving patients with rheumatic diseases and controls, such as the same pandemic COVID-19 curve in each city and region [41]. Therefore, after initial contact (V1), two strategies were adopted to maintain the follow-up of patients and to optimize the capture of main outcomes. Firstly, an active strategy is addressed in cases of flu-like syndrome and symptoms suggestive of COVID-19. For this strategy, the patient or household member is shown how to provide clinical data on the patient or his/her control through a toll-free telephone number (the 0800 system). Secondly, the patient is contacted every week by the REDCap team to gather relevant data on infection. An additional visit is conducted if the patient or control provides information about flu symptoms or disease activity via an unscheduled phone call or contact through the toll-free number. In both cases the individual will be advised to stay home and use painkillers or an antipyretic. In case of worsening/severe symptoms (eg, persistent cough or fever and shortness of breath), they will be advised to visit a hospital or to make an appointment with a physician involved in our study.

### Outcomes

Primary outcomes (at baseline and during the 24-week study period) are as follows:

- SARS-CoV-2 infection (illness): suspected, suggestive, or confirmed
- Death
- Hospitalization
- Need for intensive care unit
- Need for mechanical ventilation
- Total hospitalization time
- Date of occurrence of an adverse event (eg, death, hospitalization)

Secondary outcomes are as follows:

- General clinical differences in COVID-19 course severity between rheumatic patients and nonrheumatic controls;
- Initial clinical differences and progression to moderate or severe course of disease between the two groups.

Confounding and adjustment variables are age, sex, comorbidities, concomitant medications, and flu vaccine.

### Ethics

This project was registered with the Brazilian Registry of Clinical Trials (ID RBR-9KTWX6).

The project, which is currently in the data collection phase, was approved by the Brazilian Committee of Ethics in Human Research–CONEP (CAAE 30246120.3.1001.5505).

### Statistical Analysis

The data will be analyzed using descriptive statistics—absolute and relative frequencies for categorical variables and quantitative measures (mean, quartiles, minimum, maximum, and standard deviation) for numerical variables. The normality of the data will be verified using the Kolmogorov-Smirnov test.

A chi-square test will be used to assess the association between categorical variables with standardized adjusted residual calculation; Fisher exact test will be used for small samples. The linear associations between two variables of a numerical nature will be evaluated using Pearson correlation.

To evaluate the behavior of clinical variables between two points in time by group, analysis of variance (ANOVA) will be used, with repeated measures. In the case of nonnormality of data, the means of the groups at each time point will be compared using the Kruskal-Wallis nonparametric test. To compare the means between phone visits in each group, the Wilcoxon nonparametric test will be used.

The comparison between the means of numerical variables with normal distribution will be verified through the Student *t* test. If the assumption of normality is violated, the Mann-Whitney nonparametric test will be used.

Adjusted multiple linear regression models will be used to assess the simultaneous effects of sex, age, duration of illness, comorbidities, concomitant medications, and other confounding variables, according to group and predefined outcomes. For dichotomous dependent variables, a logistic regression model will be preferred. Survival analysis models, including log rank and Kaplan-Meier tests, adjusted for confounding variables, will be developed to assess the main outcomes over time. The time defined as the end date will be the date of a major event, such as illness with confirmation or suspicion of infection, hospitalization, or death.

Correlation analysis will be performed using the Pearson test to assess the relationship between the incidence rate of confirmed COVID-19 cases per 100,000 inhabitants and the proportion of symptomatic patients and controls in municipalities. Data of confirmed cases per municipality are available through the Brasil.io project [51]. The QGIGS Desktop 3.6.0 (Open Source Geospatial Foundation) will be used for map plotting.

SPSS, version 20 (IMB Corp) will be used in all analyses, and a *P* value <.05 will be considered significant.

## Results

This study is in the data collection phase. Study recruitment opened in March 2020. In an 8-week period (from March 29 to May 17), a total of 10,443 individuals enrolled at baseline (including 5166 patients with rheumatic diseases) from 23 tertiary rheumatology centers in 97 Brazilian cities. Data analysis is scheduled to start after all relevant data have been collected.

## Discussion

This study’s novel design, with its large sample of chronic antimalarial users, will enable us to perform a thorough prospective assessment (every 2 weeks) to explore whether vulnerability to SARS-CoV-2 infection may be associated with IMRD or to hydroxychloroquine after adjustments for cofounders, especially those related to other immunosuppressive drugs and comorbidities. On the other hand, it has some limitations, such as compliance and adherence; a potential shortage of HCQ (hydroxychloroquine) in some parts of our country; possible biases associated with recalling symptoms; inability to answer the phone (due to hospitalization or death), and lack of confirmatory tests (PCR tests for SARS-CoV-2 or antibody-based RNA [ribonucleic acid]) for all enrolled patients and controls, especially in those with nonsevere forms. Future randomized controlled trials may provide a better understanding of these differences.

## Appendix

Multimedia Appendix 1 Data collection form.

## Abbreviations

ANOVA: analysis of variance
CONEP: Brazilian National Ethics Committee
IL-6: interleukin 6
IMRD: immune-mediated rheumatic diseases
MERS-CoV: Middle East respiratory syndrome coronavirus
PCR: polymerase chain reaction
RNA: ribonucleic acid
SARS-CoV: severe acute respiratory syndrome–associated coronavirus
V: visit
