# Supplementary material for: Impact of Chronic Use of Antimalarials on SARS-CoV-2 Infection in Patients With Immune-Mediated Rheumatic Diseases: Protocol for a Multicentric Observational Cohort Study
Source: JMIR Res Protoc. 2020 Oct 14;9(10):e23532. doi: 10.2196/23532 (PMC7575340; doi:10.2196/23532)
Supplement: Multimedia Appendix 1 [file resprot_v9i10e23532_app1.pdf]

APPENDIX I

DATA COLLECTION FORM

Date: ___/____/________	Interviewer: __________________________
ID center: ______________	ID patiente: __________________________

Are you using chloroquine or hydroxychloroquine for more than 30 day?	1.Yes ( ) 2.No ( )

If not, interrupt the questionnaire and do not interview the contacts.

1. Sociodemographic data

1.1. Name:		1.2. Date of birth:
1.3. Phone:	1.4. City:	1.5. Age:
1.6. Referred weight:		
1.7. Hospital where you follow up:		
1.8. Sex: 1.F ( ) 2.M ( ) 3.Other/unknown ( )	1.9. Degree of education: 1. Unlettered( ) 2. Elementary School( ) 3. High school( ) 4. University education( )	
1.10. Profession:	1.11. Work situation: 1.active ( ) 2.Inactive ( )	

1.11. How many people live in your house?

1. Alone ( )	2. 1 Person ( )	3. 2 or 3 ( )	4. 4 or more ( )

2. About the antimalarial in use:

2.1. Which antimalarial drug is currently in use?

1. Hydroxychloroquine 400 mg ( ) 2. Chloroquine diphosphate 150 mg ( ) 3. Chloroquine diphosphate 200 mg ( )

4. Chloroquine diphosphate 250 mg ( ) 5. Chloroquine (base salt) 150 mg ( )

2.2. Usage time: ________________ months	2.3. Frequency (days of use / week)<br>1. 7 days / week ( )<br>2. 6 days / week ( )<br>3. 5 days / week ( )<br>4. 4 days / week ( )<br>5. 3 days / week ( )	2.3.1 Acquisition (dosage _______ mg)<br>1. Pharmacy purchase ( )<br>2. SUS ( )<br>3. Manipulated ( )<br>4. Others ( )

2.4 Rheumatic disease for which the antimalarial was indicated:

2.4.1. Systemic lupus erythematosus ( )	2.4.2. Systemic sclerosis ( )	2.4.3. Rheumatoid arthritis ( )
2.4.4. Cutaneous lupus ( )	2.4.5. Dermato / polymyositis ( )	2.4.6. Osteoarthritis ( )
2.4.7. Sjogren's syndrome ( )	2.4.8. Mixed Connective Tissue Disease ( )	2.4.9. Chikungunya (cronic) ( )
Other :		

2.5. Comorbidities

2.5.1 Systemic arterial hypertension	1.Yes ( )<br>2.No ( )	2.5.2 Diabetes	1.Yes ( ) 2.No ( )	2.5.3 Lung disease	1.Yes ( ) 2.No ( )
2.5.4. Heart disease	1.Yes ( )<br>2.No ( )	2.5.5. Kidney disease	1.Yes ( ) 2.No ( )	2.5.6. Others. Which one?	
2.5.7. Current smoking	1. Yes ( ) 2. No ( )				

2.5.8. Use of alcohol	1. Yes ( ) 2. No ( )		
2.6. Drugs used in combination with antimalarial OR OF INTEREST:			
2.6.1. Oral corticosteroids	1. Yes ( ) 2. No ( )	2.6.1.1 Pulse therapy with metiprednisolone	1. Yes ( ) 2. No ( )
2.6.2. Cyclophosphamide	1. Yes ( ) 2. No ( )	2.6.2.1. Pulse therapy with cyclophosphamide	1. Yes ( ) 2. No ( )
2.6.3. Mycophenolate mofetil	1. Yes ( ) 2. No ( )	2.6.4. Sulfasalazine	1. Yes ( ) 2. No ( )
2.6.5. Azathioprine	1. Yes ( ) 2. No ( )	2.6.6. Anti-TNF	1. Yes ( ) 2. No ( )
2.6.7. Cyclosporine	1. Yes ( ) 2. No ( )	2.6.8. Tofacitinib	1. Yes ( ) 2. No ( )
2.6.9. Rituximab	1. Yes ( ) 2. No ( )	2.6.10. Tocilizumab	1. Yes ( ) 2. No ( )
2.6.11. Belimumab	1. Yes ( ) 2. No ( )	2.6.12. Leflunomide	1. Yes ( ) 2. No ( )
2.6.13. Anti-inflammatory	1. Yes ( ) 2. No ( )	2.6.14. Abatacept	1. Yes ( ) 2. No ( )
2.6.15. Methotrexate	1. Yes ( ) 2. No ( )	2.6.16. BRA	1. Yes ( ) 2. No ( )
2.6.17. Angiotensin-converting enzyme inhibitors	1. Yes ( ) 2. No ( )		
2.6.1.2 Oral corticosteroid dose	1.≤5mg/dia ( )<br>2. 6 a 10 mg/dia ( )<br>3. > 10 a 20 mg/dia ( )<br>4. > 20 mg/dia ( )<br>5. Do not use ( )	2.6.15. 1 Dosage of Methotrexate	1.≤5mg/dia ( )<br>2. 6 a 10 mg/dia ( )<br>3. > 10 a 20 mg/dia ( )<br>4. > 20 mg/dia ( )<br>5. Não toma ( )
2.7. Was the antimalarial drug suspended during the epidemic? 1. Yes ( ) 2. No ( )			
2.8. If yes, how long (days)?			

**3. Epidemiological Information**			
3.1. Had contact with any suspected or confirmed case of coronavirus infection? 1. Yes ( ) 2. No ( ) 3. Don't Know ( )			
3.2. If so, where did that contact occur?			
1. During trip to endemic area ( )	2. At home ( )	3. At work ( )	4. Unknown ( )
Have you had or are you infected with coronavirus? 1. Yes ( ) 2. No ( )			

Continue only if the answer is yes. If the answer is no, go to item 6.

**4. Characteristics of coronavirus infection**			
4.1 Did you have symptoms suggestive of coronavirus infection?? 1. Yes ( ) 2. No ( )			
4.2 If you HAD SYMPTOMS, indicate which:			
4.2.1 Fever	1.Yes ( ) 2.No ( )	4.2.2 Headache	1.Yes ( ) 2.No ( )
4.2.3 Cough	1.Yes ( ) 2.No ( )	4.2.4 Dizziness	1.Yes ( ) 2.No ( )
4.2.5 Dyspnea	1.Yes ( ) 2.No ( )	4.2.6 Diarrhea	1.Yes ( ) 2.No ( )
4.2.7 Coryza	1.Yes ( ) 2.No ( )	4.2.8 Nausea	1.Yes ( ) 2.No ( )
4.2.9 Asthenia	1.Yes ( ) 2.No ( )	4.2.10 Vomit	1.Yes ( ) 2.No ( )
4.2.11 Sore throat	1.Yes ( ) 2.No ( )	4.2.12 Loss of smell	1.Yes ( ) 2.No ( )
4.2.13 Decreased taste	1.Yes ( ) 2.No ( )		
4.2.14 Others (please describe):			
4.3. Symptom onset date:			
4.4. Duration of symptoms:_______ days (if you have no symptoms, put 0)			
4.5. Still showing symptoms: 1. Yes ( ) 2. No ( ) 3. Had no symptoms ( )			
4.6. Hospital internment: 1. Yes ( ) 2. No ( )		Date of admission: ___/____/________<br>Date of hospital discharge:___/____/________	
4.6.1 Emergency Room ( ) Infirmary ( ) Intensive care unit ( )			
4.6.2 Did you need a breathing apparatus?? 1. Yes ( ) 2. No ( )			
4.7. Death 1. Yes ( ) 2. No ( ) Data of death: ___/____/________			

5. Laboratory Alteration:

5. Was the diagnosis confirmed by the PCR test?: 1. Yes ( ) 2. No ( ) 3. Unrealized ( ) 4. Others ( )

6. Assessment of rheumatic disease

6.1. Was your rheumatic disease well controlled before the coronavirus epidemic?

1. Yes ( ) 2. Partially ( ) 3. No ( ) 4. I don't Know ( )

6.2 Were symptoms worsening after coronavirus infection?

1. Yes ( ) 2. No ( ) 3. I can't correlate to the infection

6.3 Was there a change in treatment after the onset of flu-like symptoms?
1.Yes ( ) 2. No ( ) 3. I had no symptoms ( )

6.4. If yes, indicate which change
1. Dose of the drug ( ) 2. Drug suspension ( ) 3. Therapeutic scheme ( )

7. Contact information

7.1 How many people have had or are infected with coronavirus in your home?

Contact 1 and 2

7.2. Have you had any contact with a suspected or confirmed case of coronavirus infection? 1. Yes ( ) 2. No ( ) 3. Don't Know ( )

7.3. If so, where did that contact occur?

1. During trip to endemic area ( ) 2. At home ( ) 3. At work ( ) 4. Unknown ( )

7.4 If the contact was at home, what are the symptoms?

7.4.1 Fever	1.Yes ( ) 2.No ( )	7.4.2 Headache	1.Yes ( ) 2.No ( )
7.4.3 Cough	1.Yes ( ) 2.No ( )	7.4.4 Dizziness	1.Yes ( ) 2.No ( )
7.4.5 Dyspnea	1.Yes ( ) 2.No ( )	7.4.6 Diarrhea	1.Yes ( ) 2.No ( )
7.4.7 Coryza	1.Yes ( ) 2.No ( )	7.4.8 Nausea	1.Yes ( ) 2.No ( )
7.4.9 Asthenia	1.Yes ( ) 2.No ( )	7.4.10 Vomit	1.Yes ( ) 2.No ( )
7.4.11 Sore throat	1.Yes ( ) 2.No ( )	7.4.12 Loss of smell	1.Yes ( ) 2.No ( )
7.4.13 Decreased taste	1.Yes ( ) 2.No ( )		

7.4.14 Others (please describe):

7.5. Symptom onset date:

7.6. Duration of symptoms:_______days (if you have no symptoms, put 0)

7.7. Still showing symptoms: 1. Yes ( ) 2. No ( ) 3. Had no symptoms ( )

7.8. Hospital internment 1. Yes ( ) 2. No ( )
Date of admission: ___/____/________
Date of hospital discharge:___/____/________

7.8.1 Emergency Room ( ) Infirmary ( ) Intensive care unit ( )

7.8.2 Did you need a breathing apparatus? 1. Yes ( ) 2. No ( )

7.8.2 Death 1. Yes ( ) 2. No ( ) Data of death: ___/____/________
